# SkyMap: a generative graph model for GNN benchmarking

**DOI:** 10.3389/frai.2024.1427534

**Published:** 2024-11-14

**Authors:** Axel Wassington, Raúl Higueras, Sergi Abadal

**Affiliations:** Department of Computer Architecture, Universitat Politècnica de Catalunya, Barcelona, Spain

**Keywords:** Graph Neural Network (GNN), machine learning datasets, graph generation model, mixing matrix, degree distribution, benchmark

## Abstract

Graph Neural Networks (GNNs) have gained considerable attention in recent years. Despite the surge in innovative GNN architecture designs, research heavily relies on the same 5-10 benchmark datasets for validation. To address this limitation, several generative graph models like ALBTER or GenCAT have emerged, aiming to fix this problem with synthetic graph datasets. However, these models often struggle to mirror the GNN performance of the original graphs. In this work, we present SkyMap, a generative model for labeled attributed graphs with a fine-grained control over graph topology and feature distribution parameters. We show that our model is able to consistently replicate the learnability of graphs on graph convolutional, attention, and isomorphism networks better (64% lower Wasserstein distance) than ALBTER and GenCAT. Further, we prove that by randomly sampling the input parameters of SkyMap, graph dataset constellations can be created that cover a large parametric space, hence making a significant stride in crafting synthetic datasets tailored for GNN evaluation and benchmarking, as we illustrate through a performance comparison between a GNN and a multilayer perceptron.

## 1 Introduction

Graph Neural Networks (GNNs) have seen an exponential growth in interest and performance in the last years owing to their ability to model and learn from graph-structured relational data. As a result, GNNs have found their way into a broad variety of domains (Keramatfar et al., 2022) including drug discovery (You et al., 2018), Pinterest's recommendation systems (Ying et al., 2018), Google Maps' traffic prediction (Derrow-Pinion et al., 2021), or DeepMind's GraphCast climate forecast modeling (Lam et al., 2023), among many others.

An analysis of the multiple and ever appearing applications of GNNs will show that their respective datasets are markedly different not only in terms of the graph structure, but also the feature and class distributions over the graph.

In spite of this dataset variability, the availability of publicly accessible high-quality graph-structured data remains limited, particularly in comparison to the ample datasets prevalent in other deep learning modalities like images (Deng et al., 2009) or textual data (Raffel et al., 2023). Many studies in GNNs rely on datasets such as the Open Graph Benchmark (OGB) (Hu et al., 2020), which, albeit valuable, are constrained and biased toward a small set of applications. This limitation severely hampers the research community's ability to benchmark new GNN developments across the diverse range of graphs found in real-world applications.

To tackle the scarcity of public real-world graph datasets, one potential solution involves the creation of synthetic graph datasets (Palowitch et al., 2022). This approach would facilitate the generation of sizable datasets for method testing (Wassington and Abadal, 2022). For synthetic benchmarks to be effective, however, it is imperative to ensure that the generated graph datasets exhibit shapes, structures, feature distributions, and class distributions similar to those found in real-world data. As an example, real-world social networks often display community structures and occasionally adhere to scale-free characteristics (Onnela et al., 2007). Therefore, the methodologies employed for graph generation need to capture these specific properties to retain the expressivity of the resulting datasets.

Moreover, for GNN benchmarking, a crucial aspect that synthetic graph datasets must encapsulate is learnability. The generated graphs should perform comparably to real-world graphs when processed by GNNs to be of utility. Failing to mimic the behavior of real graphs would render the generated graphs unusable, as they will not provide informative insights into the mechanisms that make GNNs work for particular types of datasets.

Despite the extensive bibliography related to graph generation, many existing models (Erdos and Rényi, 2022; Watts and Strogatz, 1998; Albert and Barabási, 2002) focus on the graph structure and lack the generation of node labels and features necessary for graph learning. Moreover, many existing models, originally designed for tasks unrelated to GNNs, often prioritize mimicking characteristics that are not necessarily crucial for the specific problem at hand. For instance, while metrics like diameter and degree distribution are pertinent for understanding social networks, they may not significantly impact GNN performance. Conversely, factors such as the structure of the mixing matrix, i.e., whether nodes of two particular classes tend to be connected or not (Newman, 2003), play a more substantial role in GNN tasks but are frequently overlooked or oversimplified. Another common issue highlighted by Bonifati et al. (2020) is the lack of multi-domain applicability in existing models. For example, some models enforce specific degree distributions common in social networks but not present in other domains, such as graph representations of optimization processes where GNNs are also utilized (Schuetz et al., 2022). Even for models specifically developed for GNN benchmarking, such as ALBTER (Polina Andreeva and Bochenina, 2022), GenCAT (Maekawa et al., 2023) and Dancer (Largeron et al., 2017), achieving the desired learnability remains a challenge, as demonstrated in subsequent sections.

**Figure 1:**
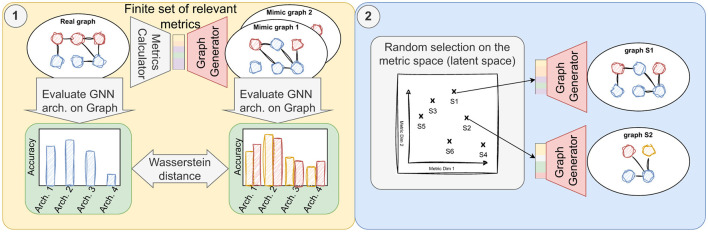
Overview of the objectives of this work. (1) Generation of synthetic graphs using metrics from real-world graph and their validation employing statistical distance measures to assess the similarity in learnability between generated graphs and their real-world counterparts. (2) Utilization of the validated generator to produce a diverse dataset, randomly sampled from points across a parametric space.

In our article, we present SkyMap^1^, a specialized generative graph model tailored to mimic the performance of GNN models, surpassing existing methods such as GenCAT and ALBTER in replicating the performance of various GNN models on real-world graphs. We benchmark our approach against three widely-used GNN architectures: Graph Convolutional Network (GCN) (Kipf and Welling, 2017), Graph Attention Network (GAT) (Veličković et al., 2018), and Graph Isomorphism Network (GIN) (Xu et al., 2019). The emulation process, shown on Figure 1, entails initially extracting a set of pertinent metrics for the node classification problem, which can be categorized into (i) metrics pertaining to class distributions and mixing matrices, i.e., the percentage of edges between nodes of different classes, (ii) metrics concerning the feature distribution, i.e., the distribution of features and their relationship with node classes, and (iii) metrics relating to graph topology, e.g. degree distribution. Subsequently, SkyMap utilizes these metrics as input to generate a graph exhibiting similar characteristics. Our evaluation reveals that SkyMap achieves a Wasserstein distance of 0.09 between the generated and real graphs, while other state-of-the-art generators typically exhibit distances exceeding 0.2. This indicates that SkyMap is more than twice as accurate as other state-of-the-art generators.

Furthermore, we delve into the practical implications of SkyMap, elucidating its novelty and presenting the obtained results. Leveraging this validated model, we curate a diverse dataset suitable for benchmarking GNN algorithms. We evaluate SkyMap based on three performance indicators: realism (being able to replicate selected real-world graphs), diversity (ensuring coverage of a sizable portion of a parametric space in the generated graphs), and utility (illustrating its use for GNN benchmarking and explainability).

The remainder of this paper is organized as follows. In Section 2, we provide a description of the state of the art in terms of synthetic graph dataset generation. In Section 3, we delve into the details of our proposed generative model SkyMap, describing the different steps of generation and the metrics used in the process. In Section 4, we describe the experiments done to assess the validity and performance of SkyMap and illustrate the significance of the proposed approach via a use case study. Finally, the paper is concluded in Section 5.

## 2 State of the art

Graph models, as statistical frameworks, play a pivotal role in capturing real graph statistics and features for analysis. Primarily generative in nature, these models enable the generation of new graphs. However, a predominant emphasis in many existing graph generators lies in modeling the graph topology, often overlooking node attributes and labels. Classical models such as the Erdos-Renyi model (Erdos and Rényi, 2022), Watts-Strogatz model (Watts and Strogatz, 1998), and Barabási-Albert model (Albert and Barabási, 2002), or the recursive matrix (R-MAT) model (Chakrabarti et al., 2004), while foundational in understanding fundamental graph properties, lack focus on modeling node-level characteristics.

The evolution of graph models traces a long history, adapting to diverse requirements over time. Initially conceived to mimic social networks, subsequent development was spurred by challenges like community detection. Notably, Stochastic Block Models emerged as a family of models specifically tailored to this objective (Lee and Wilkinson, 2019). Node classification in GNNs can be viewed as an extension of the community detection problem, integrating node features. SkyMap, introduced in this study, extends the principles of Stochastic Block Models to accommodate node attributes as well. Further, recent efforts have witnessed the advent of generative models based on deep learning approaches like VGAE (Kipf and Welling, 2016) and GraphGAN (Wang et al., 2018). However, while demonstrating prowess in tasks such as node embeddings generation, they exhibit limitations as generative graph models.

The landscape of graph modeling and benchmark dataset generation for graph algorithms remains rife with challenges. For instance, the need for graph generators with simple parameters and multi-domain applicability persists (Bonifati et al., 2020). Addressing these concerns, SkyMap employs a set of well-defined graph metrics as parameters, which enables the reproduction of graphs from diverse domains. In particular, this study focuses on generative graph models capable of modeling label and attribute distributions alongside graph topology, thereby enabling the generation of attributed and labeled graphs. Few approaches exist that can provide attributes and labels, as well as allowing some control over the graph topology (Polina Andreeva and Bochenina, 2022; Maekawa et al., 2023; Largeron et al., 2015). In the subsequent sections, we detail the characteristics of ALBTER and GenCAT, which we use as baseline graph generators in our performance evaluation.

### 2.1 ALBTER

The ALBTER model is an extension of the Block Two Level Erdos-Renyi model (BTER) (Polina Andreeva and Bochenina, 2022), that is capable of generating node labels and attributes on top of the graph structure. The BTER model (Seshadhri et al., 2011) aims to fix two of the biggest problems graph models presented when trying to model real data: capturing the heavy-tail degree distribution and maintaining the community structures. For this, the authors present a two-step approach. Firstly, ALBTER distributes the nodes in communities and connects the nodes inside each community, and after that the links between each community are created.

In ALBTER, the label assignment is extracted directly from the BTER model, assigning one different class to each of the communities, where the number of communities is an input parameter. The attribute generation is designed with the idea of having control over the attribute assortativity coefficient. For that, each node is assigned a value *X*_*v*_ = *f*_*c*_+*g*_*v*_, where *f*_*c*_~*N*(0, σ_*cluster*_) is sampled for all elements inside a cluster and *g*_*v*_~*N*(0, σ_*node*_) is sampled for each node. The variance of the distributions allow to control the assortativity as follows. If σ_*cluster*_ is large and σ_*node*_ is small, then all nodes within one cluster would be very similar but notably different from nodes in other clusters, thus giving a lower assortativity.

In their work, the authors show how ALBTER can be used for assessing the impact of certain metrics on the performance of GNNs, like the attribute assortativity or the average longest path chain. However, as we show in Section 4, modeling these characteristics well is not enough to accurately capture the performance of graphs on GCN, GAT and GIN.

### 2.2 GenCAT

GenCAT (Maekawa et al., 2023) is another graph model capable of generating labels and attributes for nodes. Contrary to ALBTER, GenCAT generates at the same time the graph topology and the features and labels. As inputs, the model needs four matrices: the mean and deviation class preference matrices, which capture how likely are different classes to be connected together; the class size distribution, which determines the size of each class; and the attribute-class correlation, which captures the relation between classes and attributes. Those input matrices are combined to generate three latent matrices, which are eventually used for the graph topology, class and attribute generation.

The model is designed to have control over two important features of real graphs: homophily and the differentiation between core and border nodes, corresponding to nodes surrounded by nodes of the same or different class, respectively. The reason for modeling homophily is that several studies have shown that such a metric has a strong influence on the performance of certain GNNs such as the GCN (Ma et al., 2022). Nevertheless, our empirical study in Section 4 shows that GenCAT is not able to capture the GNN performance with enough precision in models that do not strongly rely on homophily.

## 3 Materials and methods

### 3.1 Overview of SkyMap

**Figure 2:**
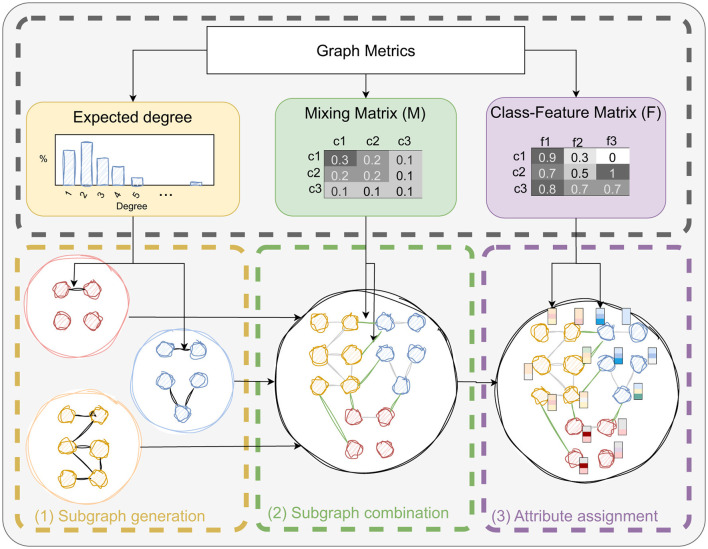
Overview of the SkyMap generative model.

Our main contribution is a novel graph model belonging to the Stochastic Block Models family. This method, which we call SkyMap, aims to generate synthetic graphs that can replicate, with high-fidelity, the behavior of real-world graphs when processed by different GNN models. To this end, SkyMap consists of four phases that are executed sequentially, as shown in Figure 2 and summarized next:

1. First, given a set of inputs relative to the graph structure, feature distribution, and class distribution, we generate the degree distributions that are later used in the graph generation. Moreover, we generate two matrices called mixing matrix *M* and class-feature matrix *F*. The mixing matrix *M* describes the probability of having edges between certain nodes based on their class, whereas the class-feature matrix *F* provides the probability of features having a certain value based on the class of the node. These methods are described in Section 3.2.
2. Then, a subgraph is generated for each class of the dataset. The number of nodes is given by an imbalance parameter describing the distribution of class populations, whereas the number of edges is given by the diagonal of the mixing matrix *M*. The placement of the edges is guided by the degree distributions also calculated in the previous step. More details are given in Section 3.3.2
3. Once all subgraphs are generated, they are connected adding inter-class edges following the distribution of the mixing matrix *M*. This is described further in Section 3.3.3.
4. Finally, once the graph topology is given, features are assigned to the nodes based on their class according to the distribution dictated by the class-feature matrix *F*. See Section 3.3.4 for more details.

The inputs that are used to generate the dataset can be obtained directly from real-world datasets or arbitrarily chosen within certain bounds. In the latter case, by executing SkyMap multiple times sampling the different input parameters accordingly, a constellation of synthetic graph datasets covering a large portion of the parametric space can be obtained. Next, the parameter set is described in more detail.

### 3.2 Model parameters

The metrics that SkyMap uses as parameters can be found, along with their bounds, in Table 1. These metrics are selected with the objective of capturing the important features that affect the accuracy of GNNs, hence providing interpretability. The final selection of metrics was obtained after an iterative process where the impact of the different metrics on the performance of different GNN variants was assessed. Next, we describe and mathematically define the different metrics, along with the methods used to reconstruct the different probability distributions or matrices required to build the graph datasets.

**Table 1:** Input parameters of SkyMap and sampling method used to generate a graph dataset constellation.

	**Parameter**	**Domain range**	**Sampling method**
Subgraph generation (imbalance distribution)	Number of nodes	[1000,1000]	Constant
	Graph density	[0, 1]	Logarithmic (10)
	Number of classes	[2, 16]	Logarithmic (2)
	Class imbalance parameter	[0, 1]	Logarithmic (10)
	Lambda (degree distribution)	[0, 10]	Uniform
	Delta (degree distribution)	[0, 50]	Uniform
Subgraph combination (mixing matrix)	Homophily	[0, 1]	Uniform
	Self affinity imbalance ratio	[0, 1]	Uniform
	Inter-class affinity imbalance ratio	[0, 1]	Uniform
	Mean class assortativity	[0, Number of classes]	Uniform
	Mean class size attraction force	[0, Number of classes]	Uniform
	Mean inverse class size attraction force	[0, Number of classes]	Uniform
	Inter-class degree multiplication mean	[0, 1]	Uniform
	Inter-class degree multiplication var	[0, mean*(1-mean)]	Logarithmic (2)
Attribute assignment (class-feature matrix)	Feature vector size (number of features)	[1, 4096]	Uniform
	Mean percentage of zeros	[0, 1]	Uniform
	Class variance	[0, mean*(1-mean)]	Uniform
	Feature variance	[0, mean*(1-mean)]	Uniform
	Inter-class dissimilarity variance	[0, mean*(1-mean)]	Uniform

We start with the definition of the main dataset parameters. The graph is defined as $G=\left(\hat{V},Ê\right)$, where $\hat{V}$ is the set of nodes or vertices, Ê is the set of edges, and is defined by $Ê\subseteq \left\{\left\{u,v\right\}|\left(u,v\right)\in {\hat{V}}^{2}\wedge v_{i}\neq v_{j}\right\}$. Then, the **number of nodes** is $n=|\hat{V}|$, whereas the **graph density** *D* is calculated as the ratio of the edges present in the graph to the maximum possible number of edges it can have, given by $D=\frac{\left|Ê\right|}{|\hat{V}|*\left(|\hat{V}|-1\right)}$. With respect to the graph connectivity, SkyMap generates subgraphs with specific degree distributions following a discrete log logistic distribution (Para and Jan, 2016), that is able to mimic both power-law and normal distributions depending on the values of its **scale parameter** λ **(Lambda)** and **shape parameter** δ **(Delta)**.

Regarding the classes, Ĉ is the set of classes, going from Ĉ_1_ to Ĉ_*k*_, such that each community Ĉ_*i*_∈Ĉ is a subset of vertices ${Ĉ}_{i}\subseteq \hat{V}$, and no vertex $v\in \hat{V}$ belongs to more than one community (Ĉ_*i*_∪Ĉ_*j*_ = ∅, ∀*i, j*∈[1, *k*]). Here, the **number of classes** is denoted as *k* = |Ĉ|. Regarding the features, let $\hat{X}$ be the matrix representing the node feature vectors, where each row ${\vec{x}}_{i}$ corresponds to the feature vector of vertex *v*_*i*_∈*V*. Thus, $\hat{X}$ is an *n*×*f* matrix, where *f* is the **number of features**.

From here, the rest of parameters refer to the generation of subgraphs using imbalance distributions (Section 3.2.1), combination of subgraphs via a mixing matrix (Section 3.2.2), and assignment of attributes by means of a class-feature matrix (Section 3.2.3).

#### 3.2.1 Imbalance distribution

One critical consideration in modeling for machine learning algorithms is the imbalance in class sizes. Extensive literature addresses strategies for handling imbalanced classes (Johnson and Khoshgoftaar, 2019), including the development of specific metrics for evaluating models trained on imbalanced data. Since real-world datasets are generally imbalanced to different extents, SkyMap should be able to generate similarly imbalanced datasets synthetically.

The objective is to find a discrete probability distribution $p_{i}^{\prime }$ that accurately represents the proportion of nodes *p*_*i*_ in each of classes *i*∈[1, *k*] where $p_{i}=|{Ĉ}_{i}|/|\hat{V}|$. This distribution can then be fitted to various graph class distributions using a maximum likelihood estimator. Subsequently, the parameters of the distribution can be utilized as metrics of the graph.

Traditionally, class imbalance has been modeled following a power-law distribution. However, power-law distributions pose two significant challenges, which we aim to address with a novel modeling approach. Firstly, power-law distributions are inherently continuous, necessitating discretization methods that may distort results depending on the application, as noted by Clauset et al. (2009). Secondly, the parameterization of power-law distributions becomes infinite for an even distribution of classes, limiting its utility.

**Figure 3:**
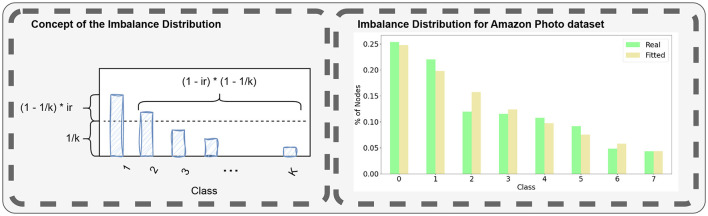
Illustration of the concept of the imbalance distribution described with Equation 1, used to generate imbalanced datasets with non-uniform class sizes. The right panel shows the class distribution of the Cora dataset and its replication using the proposed method.

To overcome these challenges, we propose an ad-hoc discrete distribution that exhibits a good fit for our validation set, as illustrated in Figure 3. This distribution is defined by the **number of classes** and the **class imbalance parameter** μ, for which low values produce rather uniform distributions (with 0 indicating perfect uniformity) while values closer to 1 yield highly imbalanced distributions. The imbalance distribution is defined as follows


(1) $$\begin{array}{lll} p_{1}^{\prime } & = & \left(1-\overset{k}{\underset{i=2}{\sum }}p_{i}\right)\\ p_{c}^{\prime } & = & \left(\frac{1}{c}+\left(1-\frac{1}{c}\right)\mu \right)\cdot \left(1-\overset{k}{\underset{j=c+1}{\sum }}p_{j}\right),\;\text{for }c\in \left[2,k-1\right]\;\\ p_{k}^{\prime } & = & \frac{1}{k}+\left(1-\frac{1}{k}\right)\mu \end{array}$$

#### 3.2.2 Mixing matrix

**Figure 4:**
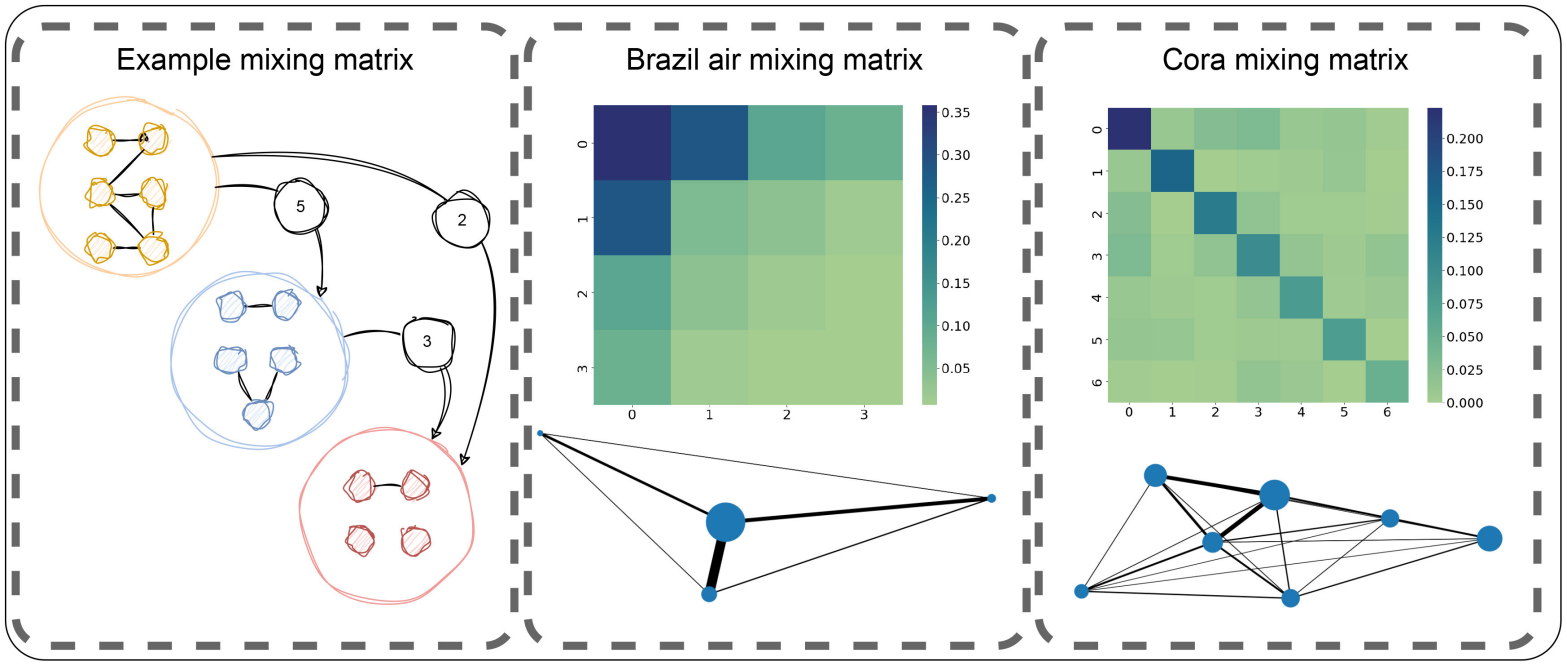
Illustration of the concept of mixing matrix as a representation of the number of edges across classes. Center and right panels represent two different mixing matrices (Brazil air and Cora) as a heatmap and as a graph where each node is a class (with its size indicating the number of edges within the class) and each edge represent the inter-class edges (with its size being the amount of edges between those classes). In this case, Brazil air has a low homophily, a high imbalance within and across classes. Instead, the Cora shows an opposite behavior with less imbalanced distributions of edges.

To model the connectivity between nodes of the same or different classes, the concept of mixing matrix is described in Newman (2003). The mixing matrix *M* is defined as a matrix of size *k*×*k*, where each row and column represents a class, and each cell value contains the proportion of edges going from one class to another ($M_{i,j}=|\left\{\left\{u,v\right\}|u\in \hat{C_{i}}\wedge v\in \hat{C_{j}}\right\}|/|Ê|$). For the case of undirected graphs, this matrix is symmetrical. Some examples of mixing matrices can be seen on Figure 4 that can help understand the different metrics discuses in the following paragraphs. Based on the mixing matrix, we define the following metrics:

- **Homophily:** $\overset{k}{\underset{c=1}{\sum }}M_{c,c}$. A high homophily value means that nodes from the same class are very connected with other nodes of the same class.
- **Self-affinity imbalance ratio:** imbalance of the values on the diagonal of *M*, computed in the same way as the class imbalance parameter, as shown in Equation 1.
- **Inter-class affinity imbalance ratio:** imbalance of the values outside of the diagonal of *M*, computed in the same way as the class imbalance parameter, as shown in Equation 1.

In addition to the previously mentioned parameters, we introduce three additional parameters aimed at fully reconstructing the mixing matrix. To achieve this, we first introduce the concept of class assortativity ($\vec{a}$). This is a novel concept inspired by the concept of graph assortativity (Newman, 2002). Class assortativity is calculated on the graph defined by the intra-class and the inter-class edges (where the intra-class edges define the weight of the nodes and the intra-class edges define the weight of the edges), as illustrated in Figure 4. Given that the classes are ordered increasingly by the number of intra-class edges, this vector is constructed in the following manner:


(2) $$\begin{array}{l} {\vec{a}}_{i}=\underset{j,j^{\prime }:j-j^{\prime }=i}{\sum }M_{j,j^{\prime }} \end{array}$$

This vector codes information about the how the edges are distributed with respect to the classes' sizes. Higher values on the last elements of the vectors indicate that the bigger classes are highly connected with the smaller classes.

Additionally, we define the class size attraction force vector $\vec{g}$ where each element *i*∈[1, *k*] is the number of connection a class has with classes of smaller size:


(3) $$\begin{array}{l} {\vec{g}}_{i}=\overset{k}{\underset{j=i+1}{\sum }}M_{i,j} \end{array}$$

This vector encodes information regarding the attraction or rejection of classes with high number of intra-class edges toward inter-class connections. We also define the inverse class size attraction force vector (*g*^†^), which is defined exactly as *g* but exchanging rows for columns, which complements the information from $\vec{g}$. Using these three vectors, we finally define the following metrics:

- **Mean class assortativity:** Weighted average of the *a* index, using the values of *a* as weights ($\overset{k}{\underset{i=1}{\sum }}i*{\vec{a}}_{i}$). This indicates the average distance in terms of class size for inter-class connections. A high value indicates that there are many connections between classes of different sizes, while a low value indicates that most of the connections happen between classes closer in size.
- **Mean class attraction force:** Weighted average of the *g* index, using the values of *g* as weights ($\overset{k}{\underset{i=1}{\sum }}i*{\vec{g}}_{i}$). This indicates the average distance in terms of class size for inter-class connections, accounting only for connections from larger to smaller classes. A high value indicates that the larger classes concentrate most of the inter-class edges connecting to smaller classes.
- **Mean class inverse attraction force:** Weighted average of the *g*^†^ index, using the values of *g* as weights ($\overset{k}{\underset{i=1}{\sum }}i*{\vec{g^{†}}}_{i}$). This indicates the average distance in terms of class size for inter-class connections, accounting only for connections from small to larger classes. A high value indicates that the smaller classes concentrate most of the inter-class edges connecting to larger classes.

#### 3.2.3 Class-feature matrix

Despite SkyMap being general, for simplicity we considered only binary features in this paper (*X*_*i, j*_∈{0, 1}). Being the features binary and in order to understand the variation of the feature behavior across classes, we define the Class-Feature matrix *F* of size *k*×*f*. Each element of the matrix is the percentage of zeros that a feature has for nodes belonging to a specific class (*F*_*i, j*_ = |{*X*_*u, j*_|*u*∈Ĉ_*i*_∧*X*_*u, j*_ = 0}|/|Ĉ_*i*_|). Then, we define four parameters that characterize the feature distribution, namely:

- **Mean percentage of zeros:** The average percentage of zeros in each class (*Mean*({*Mean*({*F*_*i, j*_, ∀*j*}), ∀*i*})). A low value indicates that the feature matrix is sparse and contains a high number of zeros. This parameter can be generalized to non-binary feature vectors.
- **Class variance:** The average of the variance value of each feature (*Mean*({*Var*({*F*_*i, j*_, ∀*j*}), ∀*i*})). A low value indicates that the classes have similar feature distributions.
- **Feature variance:** The variance of the average value of each feature (*Var*({*Mean*({*F*_*i, j*_, ∀*j*}), ∀*i*})). A low value indicates that the features are similar among the classes.
- **Inter-class dissimilarity variance:** The variance of the variance of the values of each feature (*Var*({*Var*({*F*_*i, j*_, ∀*j*}), ∀*i*})). A low value indicates that the feature distributions for each class behave similarly.

### 3.3 Dataset generator

Here, we detail the steps followed to generate the actual graph datasets, involving the reconstruction of the mixing and class-feature matrices, subgraph generation, subgraph combination, and attribute assignment.

#### 3.3.1 Matrix reconstruction

Before the actual graph generation, SkyMap performs an important pre-computation: generating the mixing matrix of the graph to be created. To generate the mixing matrix *M*, we use seven input parameters described above: the number of classes (*p*_*k*_), homophily (*p*_*h*_), self-affinity imbalance (*p*_self-aff-imb_), inter-class affinity imbalance (*p*_inter-aff-imb_), mean class attraction force (*p*_att-forc_), mean class inverse attraction force (*p*_att-forc-inv_) and mean class assortativity (*p*_class-assort_). The exact generation method can be seen in Algorithm 1. The reconstruction of the class-feature matrix is also done in this step, but explained in subsequent sections for clarity.

**Algorithm 1:**
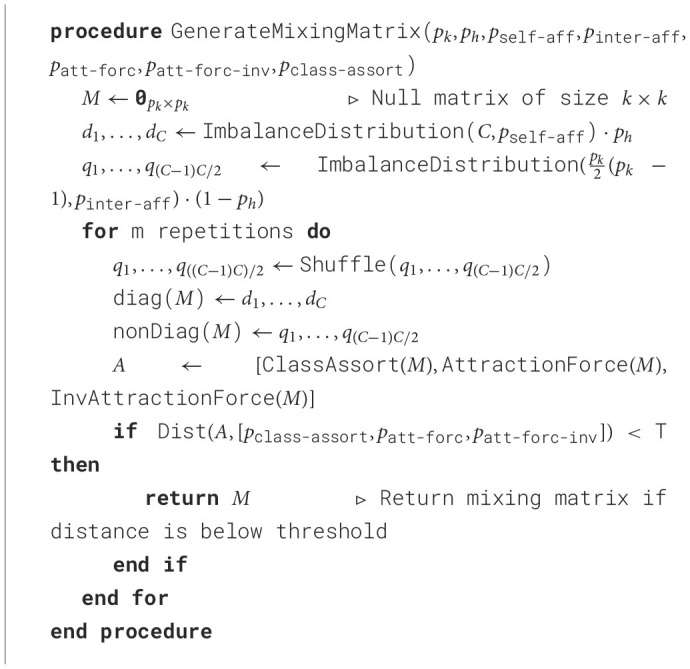
Mixing matrix reconstruction.

#### 3.3.2 Subgraph generation

Once the mixing matrix is computed, SkyMap generates one subgraph per each of the target classes. In order to determine the number of nodes of each class, a distribution of nodes is generated using the input imbalance parameter (*p*_μ_) and the total number of nodes (*p*_*n*_) as described in Section 3.2.1. The number of edges of each class is determined directly by the mixing matrix (*M*) and the density (*p*_*d*_), using the diagonal entries, and the total number of edges that is calculated using the total number of nodes and the density of the graph. Then, for each class, a graph is generated with a specific degree distribution following a discrete log logistic distribution (Para and Jan, 2016) using the Lambda *p*_λ_ and Delta *p*_δ_ parameters of the distribution. This process can be seen in Algorithm 2.

**Algorithm 2:**
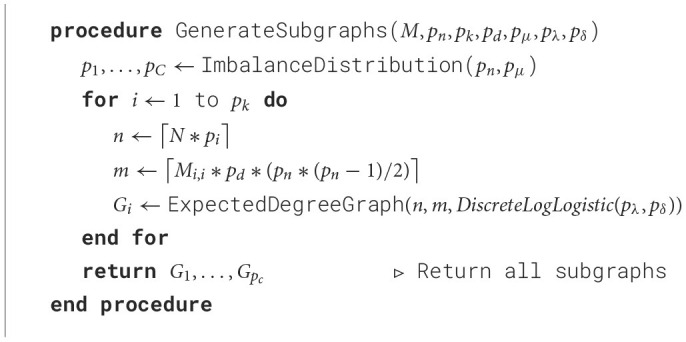
Subgraph generation.

#### 3.3.3 Subgraph combination

In this step, all the subgraphs generated in the previous step are combined into a single graph. To accomplish that, we add edges in between the subgraphs to make the entire graph connected. The number of edges added between each pair of subgraphs is determined by the mixing matrix *M*, and the edges are selected using a custom approach based on the distribution of the degree multiplication of the pairs of nodes belonging to different groups following a beta distribution with mean *p*_dmm_ and variance *p*_dmv_. This algorithm is shown in Algorithm 3.

**Algorithm 3:**
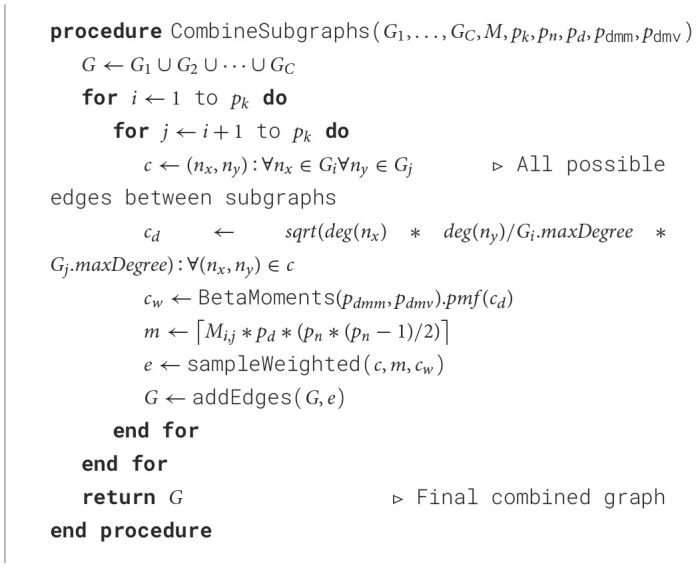
Subgraph combination.

#### 3.3.4 Attribute assignment

Finally, a feature vector is created for each node, which can be represented as a feature matrix. In order to generate the feature matrix, we use five input parameters: the number of features (*p*_*f*_), the average percentage of zeros (*p*_*mm*_), the class variance (*p*_*mv*_), the feature variance (*p*_*vm*_) and the inter-class dissimilarity variance (*p*_*vv*_). Those parameters are used as input for beta distributions using the moments method to generate the matrix *F* as defined in Section 3.2.3. Finally, the content of the feature matrix *X* is generated by sampling a Bernoulli distribution for each node and parameter, using the values in *F* as the probability parameter. This method allows SkyMap to generate node distributions that behave similarly inside classes and in between classes. This way, the generated graph is able to generate classes with very similar or dissimilar nodes, independently for each class. The resulting routine is outlined in Algorithm 4.

**Algorithm 4:**
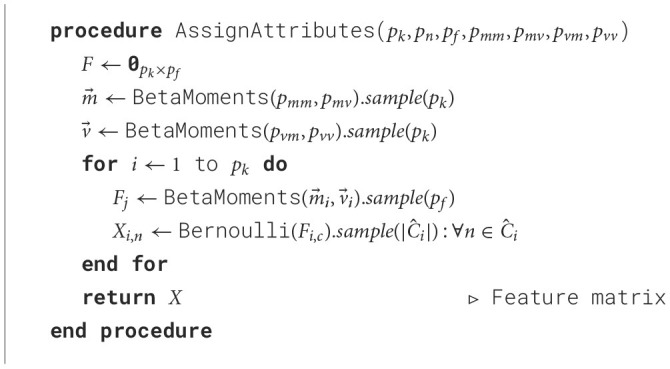
Attribute assignment.

## 4 Results

We have developed three experiments to demonstrate the realism, diversity, and utility of the developed approach for GNN benchmarking. We first validate the generator in Section 4.1 by comparing it to other state-of-the-art generators and showing that we obtain a much closer (i.e., realistic) approximation to the behavior of real graphs. Second, in Section 4.2, we build a constellation of graphs using the generator and show that this comprehensive dataset meets the diversity requirement of benchmarking. In Section 4.3, we show the utility of SkyMap by illustrating a practical usage of the generated dataset. In particular, we show that SkyMap can be used for GNN model selection and to understand the behavior of GNNs for different graph types. Finally, in Section 4.4 we assess the computational complexity of SkyMap.

For all of these experiments, we curated a validation set comprising diverse real-world graphs, ensuring binary features for consistency across models. This dataset is composed of nine well-known graphs belonging to different problems that are addressed using GNNs, as described below and in Table 2:

- **Citeseer** and **Cora**: Publication citation/coauthor networks. The task is to find the subject or tags of the publications.
- **Coauthor-Cs**: A co-authorship network. The task is to map authors to their respective field of study.
- **Amazon-photo** and **Amazon-comp**: Co-purchase networks. The task is to map goods to their respective product category.
- **Wiki Cham** and **Wiki Squirrel**: Sitemaps of Wikipedia pages related to a subject. The task is to find the most visited pages.
- **Brazil air**, **EU air**, and **USA air**: Graphs representing flight connection data. The task is to find the most visited airports.

**Table 2:** Overview of validation (real-world) dataset metrics.

**Dataset**	**# Nodes**	**# Classes**	**Density**	**Class imb**.	**Homophily**	**Class assort**.	**# Features**	**Class variance**
Citeseer	3,327	6	3e-3	0.07	0.7	3	3,703	4e-8
Cora	2,708	7	1e-2	0.12	0.8	3.1	1,433	1e-7
Coauthor-Cs	18,333	15	9e-3	0.12	0.8	5.3	6,805	8e-6
Amazon-photo	7,650	8	4e-2	0.14	0.8	5.6	745	7e-3
Amazon-comp	13,752	10	3e-2	0.2	0.7	4.4	767	4e-3
Wiki Cham	2,277	5	1e-2	0.03	0.2	2.2	2,325	2e-5
Wiki Squirrel	5,201	5	2e-2	0	0.2	2.4	2,089	2e-5
Brazil air	131	4	2e-1	0.02	0.4	1.9	131	0
EU air	399	4	1e-1	0.01	0.4	1.9	399	0
USA air	1,190	4	5e-2	0	0.7	1.8	1,190	0

All the experiments are run using the same software, the PyTorch Geometric framework (Fey and Lenssen, 2019) with CUDA version 10.1 and torch version 1.10.2; as well as the same hardware, a machine with CPU Intel(R) Core(TM) i7-2600 CPU @ 3.40GHz, GPU GeForce GTX 980 Ti and 15 GB of RAM.

### 4.1 SkyMap validation

In a first experiment, we compare SkyMap with two state-of-the-art generators (ALBTER and GenCAT). In order to use the ALBTER and GenCAT models for mimicking graphs, we followed the process present in their papers and repositories (Polina Andreeva and Bochenina, 2022; Maekawa et al., 2023). For GenCAT, the necessary input matrices are directly computed from the original graphs. Additionally, the extra attribute-class correlation matrix is generated by computing the average number of ‘1' values for each pair of attribute-class on the original graph. In the case of ALBTER, a preliminary step of parameter tuning is conducted for the sake of a fair comparison. This process involves a grid search aimed at minimizing the difference between original model metrics and the metrics obtained on the generated graphs. Specifically, the metrics considered for optimization include homophily, feature assortativity, average clustering coefficient, average shortest path length, and average degree. Following the grid search, the parameters yielding the closest overall results are selected for mimicking that specific graph.

The experiment consists in assessing the similarity between accuracy obtained when the generated graphs and the original graphs they aim to mimic are used to train a given GNN. In particular, we replicated each real-world graph three times with each generator and trained and evaluated each of the designs in our design space on each replica five times. By analyzing the resulting distributions of accuracy or F-score, we computed the Wasserstein distance to quantify the similarity of the replicated graphs from SkyMap, ALBTER, and GenCAT with those of the original real-world graph. This way, compare the performance of SkyMap with the other graph models.

Our design space comprises six possible configurations, determined by the convolutional layer type (GIN, GAT, or GCN) and the number of layers (3 or 5). All training sessions were conducted for 128 epochs, employing the ADAM optimizer with a learning rate of 1e-2. The training set consisted of 60% of the nodes, while the testing set comprised 40%. To accommodate larger graphs within GPU memory constraints, the data was partitioned for training and testing into chunks of approximately 500 nodes using the algorithm proposed by Chiang et al. (2019). This partitioning strategy enabled efficient processing of larger graphs.

**Figure 5:**
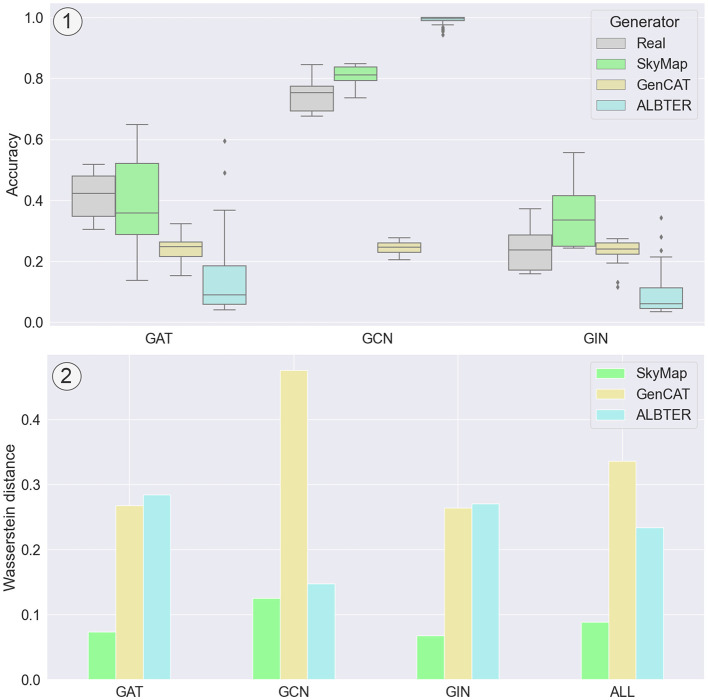
Comparison of the distribution of accuracy between real-world graphs and synthetic graphs generated with SkyMap, GenCAT, and ALBTER when trained with GAT, GCN, and GIN. (1) Results for the Amazon Photo dataset, illustrating the distribution of accuracies for each of the generators and each of the GNN models. (2) Mean Wasserstein distance for the accuracy distributions for all the considered datasets. “ALL” corresponds to the mean Wasserstein distance across GNNs as well.

As depicted in Figure 5, SkyMap demonstrates superior performance compared to the two competing generators across all considered GNN models. Notably, the closest competitor is ALBTER on the GCN model. SkyMap achieves a mean distance of 0.09 across all datasets and models for the accuracy, which is less than half the distance achieved by the other generators. A similar distance is obtained for the F1-score, with SkyMap achieving a distance of 0.08 and other generators a distance of over 0.2. This underscores the effectiveness of SkyMap in accurately mimicking the distribution of accuracy scores, highlighting its potential for generating high-fidelity graph datasets for GNN evaluation.

### 4.2 Comprehensive dataset generation

The second experiment involves generating a diverse dataset by randomly selecting points in the metric space and demonstrating how this dataset is able to cover the space (Wassington and Abadal, 2024). Specifically, we generate a dataset comprising 1000 graphs, each containing 1000 nodes. The parameters are sampled as summarized in Table 1: most parameters are uniformly sampled from the interval [0,1], some parameters follow an exponential distribution, and certain parameters depend on others. For instance, all variances of the beta distributions are bounded by the distribution of mean*(1−*mean*).

**Figure 6:**
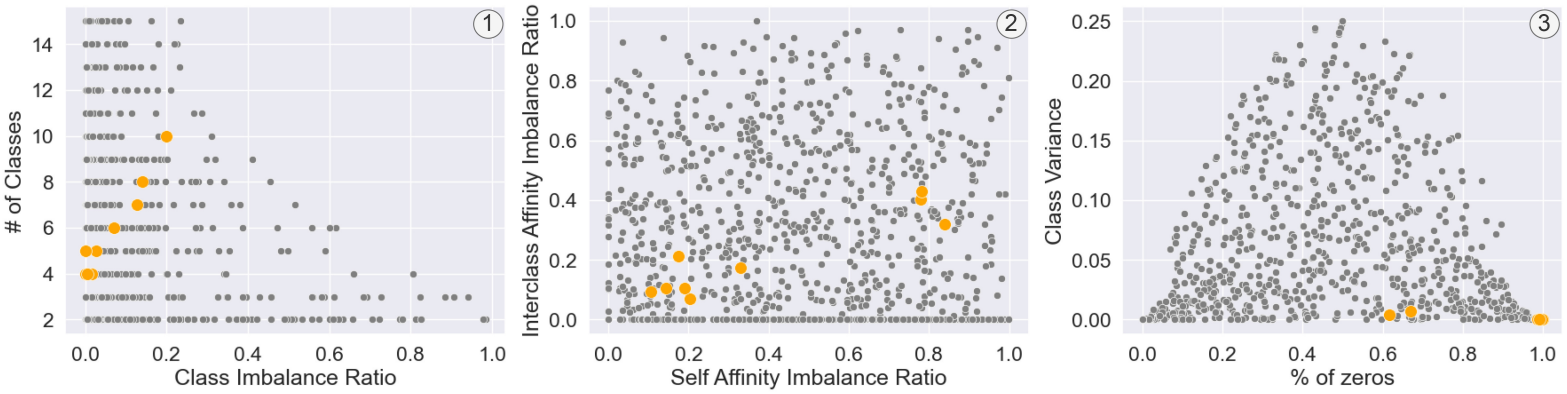
Projection of generated synthetic graphs (gray) and validation real-world graphs (orange) on six metrics involved in the different dataset generation processes, specifically (1) two metrics related to the distribution of nodes in classes (number of classes and class imbalance ratio), (2) two metrics related to the mixing matrix (interclass affinity imbalance ratio and self-affinity imbalance ratio), and (3) two metrics related to the parameter distribution (class variance and percentage of zeros).

In Figure 6, we observe that the real-world graphs are within the broad range of the parametric space delimited by the constellation of datasets generated by SkyMap. The diversity within the dataset is inherently built into its design, as the model's input parameters are the metrics intended to exhibit variation, and the sampling process ensures diversity. To evaluate the representation of real datasets, we quantify the distance between the real datasets and the closest graph from the generated dataset. This distance is then compared to the mean minimum distance between the generated graphs. If the former distance exceeds the latter by an order of magnitude, it suggests that the generated graphs fall outside the distribution. In our analysis, the distances from the validation dataset to the closest generated graph range from 0.4 to 0.7, with a mean of 0.51, while the mean minimum distance between the generated graphs is 0.45. All these distances are between normalized features. These results imply that the generated graphs effectively capture the distribution of the validation dataset.

### 4.3 Use case study

In order to showcase the utility of SkyMap, we leverage the benchmark dataset generated in the preceding sections to conduct a comparative analysis between a GNN model and a non-GNN model. Specifically, we juxtapose a Graph Attention Network (GAT) against a MultiLayer Perceptron (MLP), both employing 3 layers. Our methodology entails training and testing each graph in the generated dataset using both model architectures. Subsequently, we compute the accuracy difference between the two designs for every graph. To elucidate the nuanced interplay between various graph metrics and model performance, we employ a mixed linear + non-linear regression model. This model utilizes the same set of graph metrics employed as input for SkyMap. The model's output provides an approximation of the accuracy difference that each design is expected to achieve, which could be positive (MLP is better) or negative (GAT is better) and, hence, enables us to discern scenarios in which one design outperforms the other. Furthermore, by dissecting the impact factors, we gain invaluable insights into the influence of different metrics on the comparative performance of the two designs. To test the model we use our validation set already presented.

**Figure 7:**
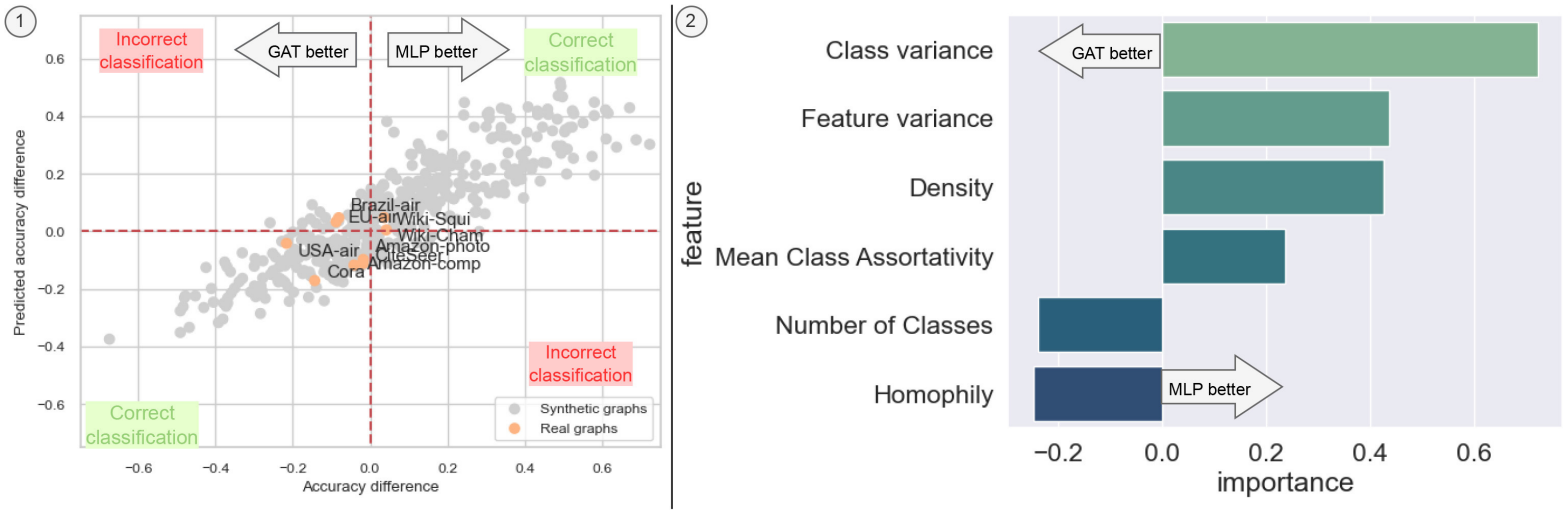
Predicted accuracy difference between the GAT model and an MLP. (1) Real vs predicted plot, showing that our model can correctly predict that a GNN is better for the evaluated real datasets, with a reasonable prediction of the difference in accuracy. (2) Feature importance of the regression.

Figure 7 illustrates how regression over the generated dataset allows us to approximate the difference in accuracy between the two models. The regression model exhibits satisfactory performance, achieving an accuracy of approximately ~77% in classifying which model performs better over our validation set, with exceptions observed for Brazil air and EU air datasets.

Notably, higher levels of homophily tend to enhance the accuracy of the GNN model compared to the MLP, as the former integrates information from the edges. However, certain factors such as density and mean class assortativity appear to introduce noise that the GAT model struggles to handle effectively. Conversely, features unrelated to graph topology, such as the variance of features between classes and across different features, are better managed by the MLP. Additionally, a higher number of classes tends to be better accommodated by the GAT model than by the MLP. Overall, these results suggest that such a regressor trained with the comprehensive graph dataset constellation of SkyMap could be useful for an *a priori* model selection based on the characteristics of the graph dataset at hand, also providing explainability of the reasons behind the selection.

### 4.4 Computational complexity assessment

**Figure 8:**
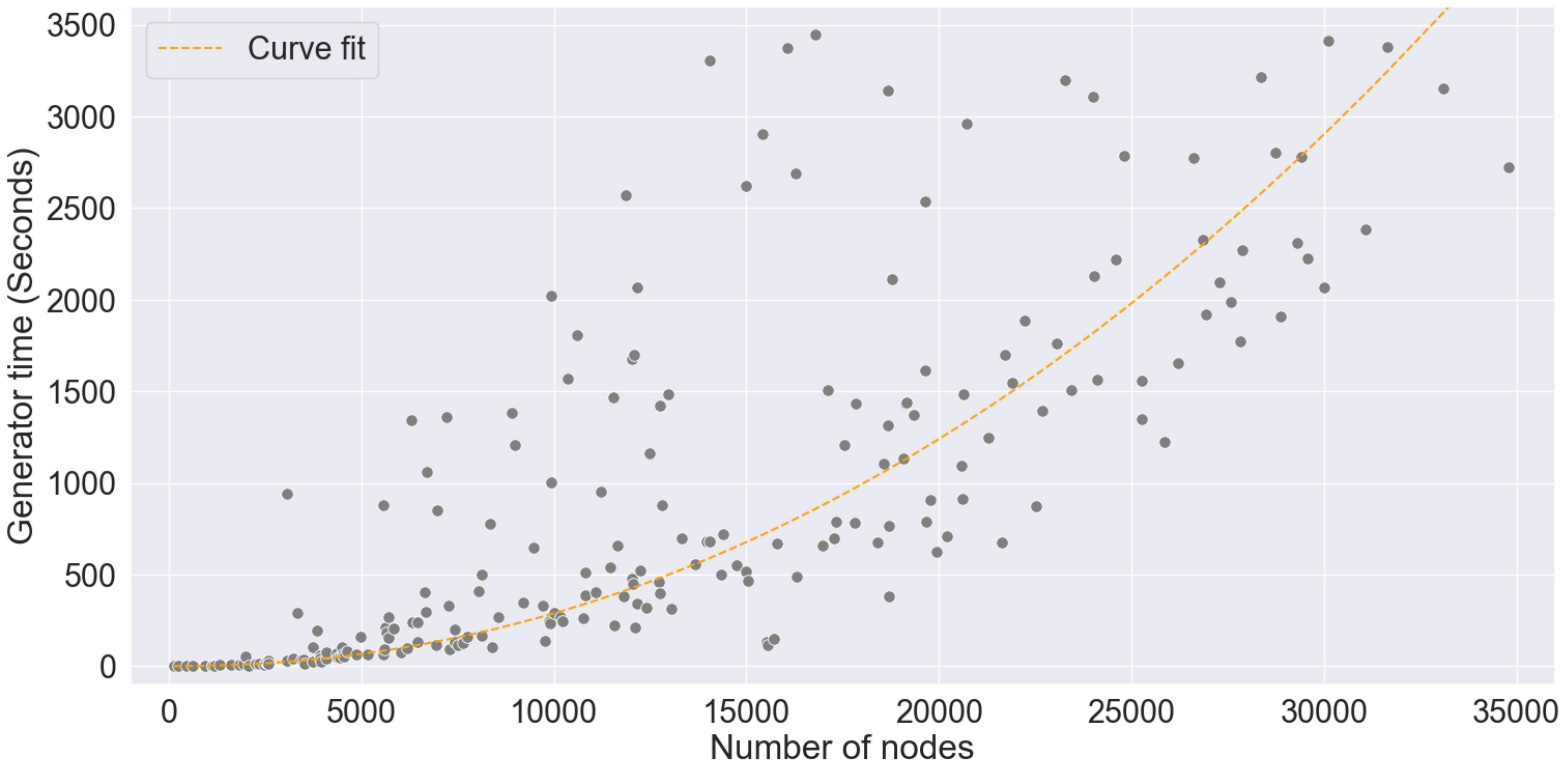
The graph generation time scales quasi-quadratically with respect to the number of nodes. In the figure, a curve is fitted to the data to demonstrate the *O*(*N*^2^log(*N*)) scaling behavior.

Finally, to study the computational complexity of the graph generator, we have registered the execution time of SkyMap under the conditions of the previous sections and also scaling the number of nodes. Figure 8 summarizes the results. With our setup, it took approximately 3 seconds to generate a graph with 1,000 nodes and around 1 hour to generate a 30,000-node graph. We can also observe that the execution time scales quadratically with the number of nodes. To clarify the origin of such a scaling trend, we analyzed the code and did some scaling tests. SkyMap's algorithm can be essentially divided in three steps, whose computational complexity scales with the number of nodes *N* and number of features *F* as follows:

- **Subgraph Generation:** The complexity is *O*(*N*^2^log*N*).
- **Subgraph Combination:** The complexity is also *O*(*N*^2^log*N*).
- **Attribute Assignment:** The complexity is *O*(*FN*log*N*).

Additionally, from the experimental results, we obtained that the subgraph combination step dominates the overall processing time, corresponding to a mean of 92% of the processing time, with the other 8% percent equally divided between the subgraph generation and the attribute assignment steps. This confirms the quadratic nature of SkyMap's execution time.

Although the graph generation process does not scale efficiently with respect to the number of nodes, it is worth noting that all the analyses conducted in the previous sections were performed with a dataset consisting of graphs of 1,000 nodes. Our results demonstrate that, even when the generated graphs differ in size from the original graphs by up to an order of magnitude, the selected metrics scale correctly with graph size and produce similar outcomes.

## 5 Discussion

In this article, we presented SkyMap, a novel graph model able to generate labeled attributed graphs seeking to solve the problem of the scarcity of large public benchmarks for GNN validation. The model was designed with the objective of capturing the attributes that affect the performance of the graph when used as an input of a GNN to solve a node classification task. We designed several experiments to showcase the usefulness and significance of the approach and to compare its performance with that of two state-of-the-art models, GenCAT and ALBTER. We showed through those experiments that our method consistently outperforms other methods on the ability to imitate the performance of the graphs on three of the most famous GNN architectures (GCN, GAT and GIN), quantified by a reduction of 64% of the Wasserstein distance of their distributions. We also showed that by randomly sampling on the input parameters of SkyMap, we can generate a graph dataset constellation covering a multi-dimensional parametric space and always having the real-world datasets within distribution. We have finally showcased the utility of SkyMap by using such a dataset constellation to train a regression model that predicts whether a GAT or an MLP will be better suited to a particular dataset just by looking at its characteristics; yet we believe that this is just one of the possible applications of the synthetic datasets generated by SkyMap.

The proposed model has proven effective in addressing node classification tasks. However, it is important to note that the model's current design does not yet directly extend to other tasks such as graph classification and edge prediction. Despite this limitation, it is plausible that with minimal modifications, the methodology could be adapted for these additional tasks. Specifically, for graph classification, the generator might be adjusted by removing the steps and metrics, e.g. homophily, that concern the distribution of classes throughout the graph and their relationship with the distribution of features and the graph connectivity. Another point for future work is an improvement of the execution time of SkyMap. In this respect, subgraph combination stands as the most time consuming step and should be analyzed further; one could assess possible parallelization strategies or analyze the impact of relaxing this step on both the time complexity of SkyMap and the quality of the generated graphs.

## Data Availability

The original contributions presented in the study are publicly available. This data can be found here: https://github.com/raulhigueras/skymap_graph_generator.

## Footnotes

^1^The code of SkyMap can be found at https://github.com/raulhigueras/skymap_graph_generator.
